# Diagnostic Value of sTREM-1 in Bronchoalveolar Lavage Fluid in ICU Patients With Bacterial Lung Infections: A Bivariate Meta-Analysis

**DOI:** 10.1371/journal.pone.0065436

**Published:** 2013-05-29

**Authors:** Jia-Xin Shi, Jia-Shu Li, Rong Hu, Chun-Hua Li, Yan Wen, Hong Zheng, Feng Zhang, Qin Li

**Affiliations:** Department of Respiratory Medicine, Lianyungang First People's Hospital, Affiliated Hospital of Xuzhou Medical College, Lianyungang, China; University of Pittsburgh, United States of America

## Abstract

**Background:**

The serum soluble triggering receptor expressed on myeloid cells-1 (sTREM-1) is a useful biomarker in differentiating bacterial infections from others. However, the diagnostic value of sTREM-1 in bronchoalveolar lavage fluid (BALF) in lung infections has not been well established. We performed a meta-analysis to assess the accuracy of sTREM-1 in BALF for diagnosis of bacterial lung infections in intensive care unit (ICU) patients.

**Methods:**

We searched PUBMED, EMBASE and Web of Knowledge (from January 1966 to October 2012) databases for relevant studies that reported diagnostic accuracy data of BALF sTREM-1 in the diagnosis of bacterial lung infections in ICU patients. Pooled sensitivity, specificity, and positive and negative likelihood ratios were calculated by a bivariate regression analysis. Measures of accuracy and Q point value (Q*) were calculated using summary receiver operating characteristic (SROC) curve. The potential between-studies heterogeneity was explored by subgroup analysis.

**Results:**

Nine studies were included in the present meta-analysis. Overall, the prevalence was 50.6%; the sensitivity was 0.87 (95% confidence interval (CI), 0.72–0.95); the specificity was 0.79 (95% CI, 0.56–0.92); the positive likelihood ratio (PLR) was 4.18 (95% CI, 1.78–9.86); the negative likelihood ratio (NLR) was 0.16 (95% CI, 0.07–0.36), and the diagnostic odds ratio (DOR) was 25.60 (95% CI, 7.28–89.93). The area under the SROC curve was 0.91 (95% CI, 0.88–0.93), with a Q* of 0.83. Subgroup analysis showed that the assay method and cutoff value influenced the diagnostic accuracy of sTREM-1.

**Conclusions:**

BALF sTREM-1 is a useful biomarker of bacterial lung infections in ICU patients. Further studies are needed to confirm the optimized cutoff value.

## Introduction

Infection is a common challenge for patients in the intensive care units (ICU). Lung is the main site of infection (including ventilator-associated pneumonia (VAP)) and the ICU-acquired pneumonia increases the risk of ICU death [1]. The best way to diagnose and appropriately treat lung infection is to identify the microorganism(s) responsible for the infections, however, the microorganism culture will delay diagnosis and subsequent antibiotics use, which is associated with worse outcomes [2]. Obviously, it is necessary to initiate urgent empirical antibiotic treatment, nevertheless, the presence of underlying diseases such as congestive heart failure, atelectasis, acute respiratory distress syndrome and pulmonary hemorrhage may misguide diagnosis. So it is critical to confirm the existence of lung infection early and accurately.

Previous studies have reported a number of biomarkers (for example, C-reactive protein (CRP) [3], procalcitonin (PCT) [3], soluble triggering receptor expressed on myeloid cells-1 (sTREM-1) [4], interleukin-1beta (IL-1β) [5], and plasminogen activation inhibitor-1 [6]) that were used to determine the diagnosis and prognosis for lung infections. Globally, sTREM-1 and PCT were considered to be promising markers for diagnosis of lung infections. However, a previous study showed PCT (with a cutoff value of 3.9 ng/ml) had a sensitivity of 41% and a specificity of 100% in diagnosis of VAP [7], and another study showed serum sTREM-1 (with a cutoff value of 3.5 µg/L) had a sensitivity of 82% and a specificity of 40% in diagnosis of bacterial infections in emergency department [8]. Recently, Ramirez et al. [9] evaluated the diagnostic accuracy of sTREM-1 in bronchoalveolar lavage fluid (BALF) in ICU patients with lung or abdominal infections and showed a sensitivity of 81% and a specificity of 80% (with a cutoff value of 900 pg/ml) in diagnosis of pneumonia, which implied that BALF sTREM-1 could be more accurate in diagnosing lung infections.

Herein, we performed a meta-analysis of eligible clinical studies to assess the accuracy of BALF sTREM-1 as a diagnostic marker for bacterial lung infections in ICU patients using a bivariate regression approach.

## Materials and Methods

### Data sources

We searched PUBMED, EMBASE and Web of Knowledge (from January 1966 to October 2012) databases for relevant studies that reported diagnostic accuracy data of BALF sTREM-1 in the diagnosis of lung infections in ICU patients. The following search terms were used: sTREM-1, soluble triggering receptor expressed on myeloid cells-1, pneumonia, infection, alveolar, bronchoalveolar, lavage, BAL (F). The search was restricted to human subjects. If a study was included in a review and was indexed in the PUBMED database, the related studies were further explored using the “Related Articles” option. We also reviewed the relevant references listed in the searched papers.

### Study selection

The studies were included if they fulfilled the fowling criteria: (1) patients were hospitalized in ICU; (2) original publication; and (3) true-positive (TP), false-positive (FP), false negative (FN), and true-negative (TN) results of the diagnostic tests for the detection of lung infections were reported or could be calculated. The studies with populations fewer than 10 were excluded in order to avoid selection bias.

### Data extraction and quality assessment

Two reviewers (Jia-Xin Shi and Jia-Shu Li) independently identified the trials and extracted the data to obtain information on the trials. Each reviewer extracted the data to construct a 2×2 table. If disagreement occurred, two reviewers discussed and arrived at consensus.

The Quality Assessment of Diagnostic Accuracy Studies (QUADAS) [10] and the Standards for Reporting of Diagnostic Accuracy (STARD) checklist [11] were used to assess the methodological quality of the included trials.

### Statistical analysis

We used a bivariate regression approach to calculate the pooled sensitivity (SEN) and specificity (SPE), positive and negative likelihood ratios (PLR and NLR, respectively), and constructed summary receiver operating characteristic (SROC) curve to summarize the study results [12]. We also calculated the respective area under the SROC curve and Q point value (Q*), where sensitivity  =  specificity, on the SROC curve.

The between-studies heterogeneity was evaluated by the I^2^ test for the pooled diagnostic odds ratio (DOR) [13]. The DOR is the ratio of the odds of a positive test result in patients with lung infections compared with patients without lung infections: (TP/FN)/(FP/TN). I^2^≥50% indicated substantial heterogeneity.

We performed subgroup analysis to explore the sources of potential heterogeneity among studies using univariate meta-regression analysis. The covariates included: Prevalence (≥50% (High) vs. <50%), Sample (BALF vs. mini-BALF), Culture Threshold (≥10^4^/CFU vs. others), Assay Method (ELISA vs. others), Cutoff (≥200 pg/ml vs. others), and Gold Standard (“clinical+radiological+microbiological” vs. “clinical+microbiological”). Publication bias were inspected by Deek's funnel plot [14]. The Fagan's nomogram was used to calculate the posttest probability (PTP) [15]. We used the STATA software, version 11 (Stata Corporation, College Station, TX) with the “Midas” module and Meta-Disc 1.4 (XI Cochrane Colloquium, Barcelona, Spain) to perform all the analysis. A *P* value < 0.05 was considered to be statistically significant.

## Results

### 1. Study characteristics

**Figure 1:**
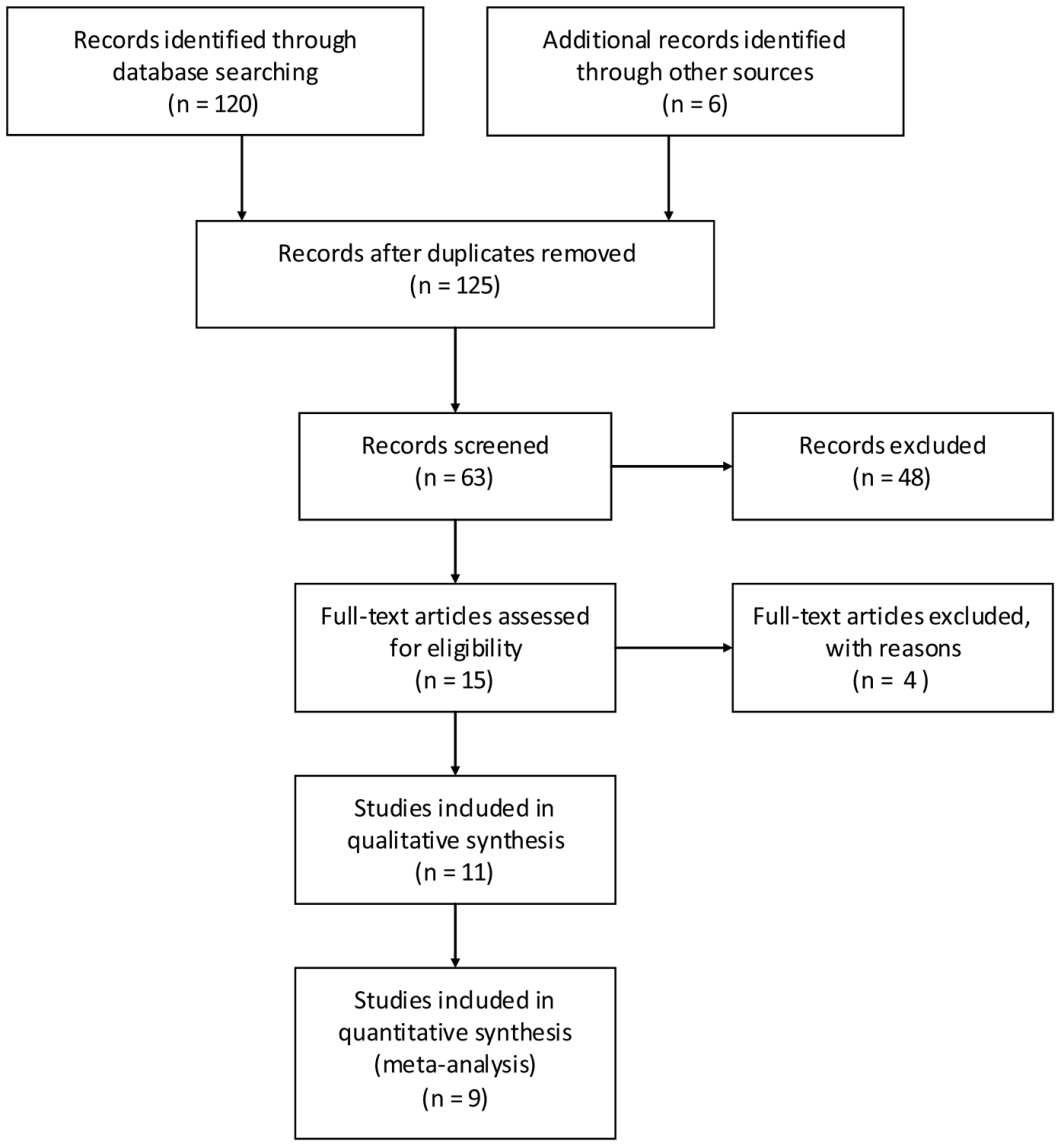
Flow diagram of identification of relevant studies.

Of 103 searched English articles and 23 Chinese articles, we excluded 117 that were not relevant to our study question on the basis of title and abstract. Four studies [16]–[19] were excluded after the full-text assessment: two [16], [18] of them compared the mean levels of BALF sTREM-1 between the pneumonia and non-pneumonia groups, however, the data could not be extracted to construct a 2×2 table; one [17] of them compared the mean levels of BALF sTREM-1 between the survivors and non-survivors; another study [19] evaluated the BALF sTREM-1 levels in children with VAP. Nine studies [4], [9], [20]–[26] with 510 patients met the inclusion criteria and were selected for the meta-analysis (Fig. 1).

The characteristics and results of the included trials were shown in Table 1. The diagnosis of bacterial lung infection was based on the clinical and radiological findings, laboratory results, Gram-stain results, and culture results. One study used “clinical+microbiological” results as gold standard for the diagnosis of bacterial lung infection, and the others used “clinical+radiological+microbiological” findings as gold standard. Mini-BALF was collected in two studies [4], [9], and BALF was collected in the other seven studies. Culture results (bacteria or fungi) were considered to be critical in the diagnosis of lung infections in all studies. Two studies used the immunoblot technique method for sTREM-1 measurements [4], [21], and the ELISA assay method was used in the other seven studies. The cutoff values ranged from 5 pg/ml to 900 pg/ml.

**Table 1:** Characteristics of studies included in the meta-analysis.

Study	Country	Gold standard	Design	Age (y)	Patients (n)	Prevalence (%)	Type of infection	Sample	Microorganism criteria (BALF culture)	Assay method	Cutoff (pg/ml)	TP	FP	FN	TN
Gibot et al, 2004 [4]	France	C+R+M	PR	≥18	148	57	CAP/VAP	mini-BALF	≥10^3^ CFU/ml	Immunoblot technique	5	82	6	2	58
Determann et al, 2005 [20]	Netherlands	C+R+M	PR	Adult	27	30	VAP	BALF	≥10^4^ CFU/ml	ELISA	200	7	3	1	16
Gibot et al, 2007 [21]	France	C+M	PR	NA	50	62	VAP	BALF	≥10^3^ CFU/ml	Immunoblot technique	5	29	2	2	17
Horonenko et al, 2007 [22]	USA	C+R+M	PR	≥18	24	58	VAP	BALF	>10^3^ CFU/ml	ELISA	7	14	9	0	1
Huh et al, 2008 [23]	Korea	C+R+M	PR	≥18	80	36	Bacterial or Fungal pneumonia	BALF	>10^4^ CFU/ml	ELISA	184	25	5	4	46
El Solh et al, 2008 [24]	USA	C+R+M	PR	NA	75	51	Pulmonary aspiration syndromes	BALF	>10^4^ CFU/ml	ELISA	250	25	3	13	34
Anand et al, 2009 [25]	USA	C+R+M	PR	Adult	40	48	VAP	BALF	>10^3^ CFU/ml	ELISA	200	8	2	11	19
Ramirez et al, 2011 [9]	Spain	C+R+M	PR	NA	21	76	VAP/HAP	mini-BALF	≥10^3^ CFU/ml	ELISA	900	13	1	3	4
Palazzo et al, 2012 [26]	USA	C+R+M	PR	>18	45	42	VAP	BALF	≥10^3^ CFU/ml, or ≥10^2^ CFU/ml of PSB culture	ELISA	204	15	20	4	6

ICU, intensive care unit; C, clinical; R, radiological; M, microbiological; PR, prospective recruitment; NA, not available; CAP, community acquired pneumonia; VAP, ventilator associated pneumonia; HAP, hospital acquired pneumonia; BALF, bronchoalveolar lavage fluid; CFU, colony-forming units; PSB, protected specimen brush; ELISA, enzyme-linked immunosorbent assay; TP, true-positive; FP, false-positive; FN, false-negative; TN, true-negative.

### 2. Quality assessment of the included studies

**Figure 2:**
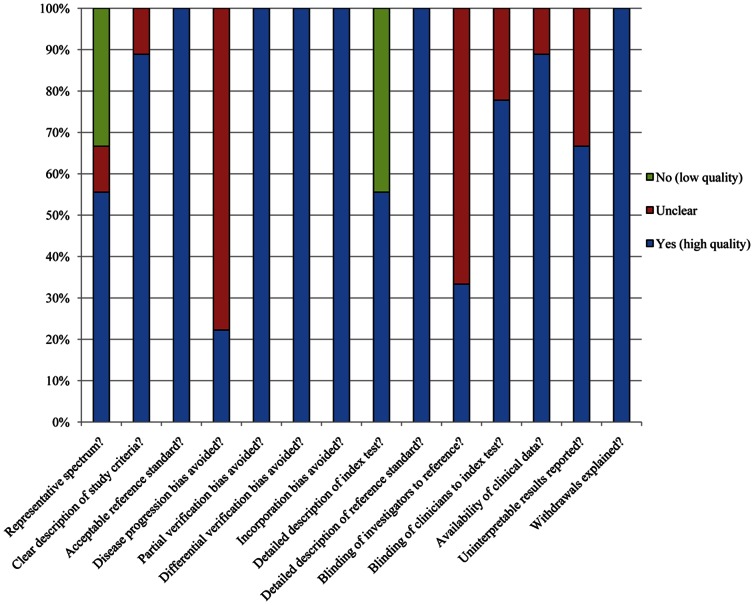
Methodological quality assessment of the included studies. Data are presented as a percentages bar across all included studies.

The methodological quality assessment for the included nine studies was shown in Table 2 and Fig. 2. All the studies included in our meta-analysis met on average 10 of the 14 QUADAS criteria, which showed high quality.

**Table 2:** QUADAS criteria for each study included.

QUADAS criteria		Gibot et al, 2004 [4]	Determann et al, 2005 [20]	Gibot et al, 2007 [21]	Horonenko et al, 2007 [22]	Huh et al, 2008 [23]	El Solh et al, 2008 [24]	Anand et al, 2009 [25]	Ramirez et al, 2011 [9]	Palazzo et al, 2012 [26]
Representative spectrum?		Y	Y	N	Y	Y	N	Y	N	U
Clear description of study criteria?		Y	Y	Y	Y	Y	U	Y	Y	Y
Acceptable reference standard?		Y	Y	Y	Y	Y	Y	Y	Y	Y
Disease progression bias avoided?		U	U	Y	U	U	Y	U	U	U
Partial verification bias avoided?		Y	Y	Y	Y	Y	Y	Y	Y	Y
Differential verification bias avoided?		Y	Y	Y	Y	Y	Y	Y	Y	Y
Incorporation bias avoided?		Y	Y	Y	Y	Y	Y	Y	Y	Y
Detailed description of index test?		Y	Y	N	Y	Y	Y	N	N	N
Detailed description of reference standard?		Y	Y	Y	Y	Y	Y	Y	Y	Y
Blinding of investigators to reference?		U	U	Y	Y	U	Y	U	U	U
Blinding of clinicians to index test?		U	Y	Y	Y	Y	Y	U	Y	Y
Availability of clinical data?		Y	Y	Y	U	Y	Y	Y	Y	Y
Uninterpretable results reported?		Y	Y	Y	Y	U	U	Y	U	Y
Withdrawals explained?		Y	Y	Y	Y	Y	Y	Y	Y	Y

Y, yes (high quality); U, unclear; N, no (low quality).

### 3. Data synthesis and meta-analysis

**Figure 3:**
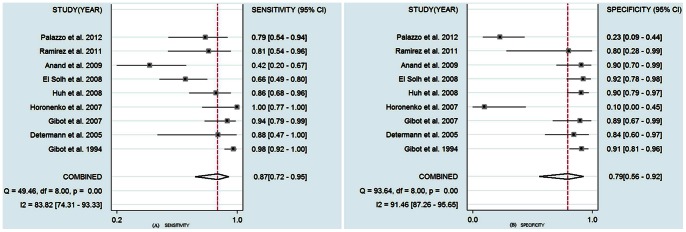
Forest plot of the sensitivity (A) and specificity (B) of BALF sTREM-1 in the diagnosis of bacterial lung infections in ICU patients. The results were as follows: sensitivity, 0.87 (95% CI, 0.72–0.95); specificity, 0.79 (95% CI, 0.56–0.92).

**Figure 4:**
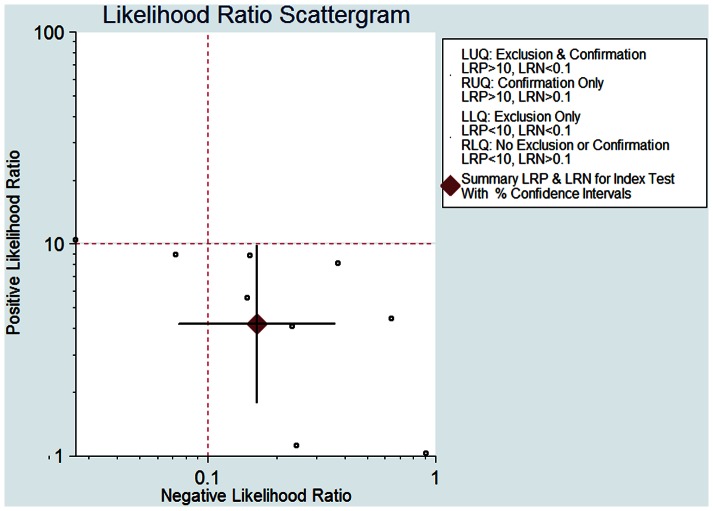
Scattergram of the positive likelihood ratio (PLR) and negative likelihood ratio (NLR). The results were as follows: PLR 4.18 (95% CI, 1.78–9.86), NLR 0.16 (95% CI, 0.07–0.36).

The pooled SEN of BALF sTREM-1 was 0.87 (95% confidence interval (CI), 0.72–0.95) (Fig. 3A) and the pooled SPE was 0.79 (95% CI, 0.56–0.92) (Fig. 3B). The PLR was 4.18 (95% CI, 1.78–9.86) (Fig. 4), the NLR was 0.16 (95% CI, 0.07–0.36) (Fig. 4), and the DOR was 25.60 (95% CI, 7.28–89.93).

**Figure 5:**
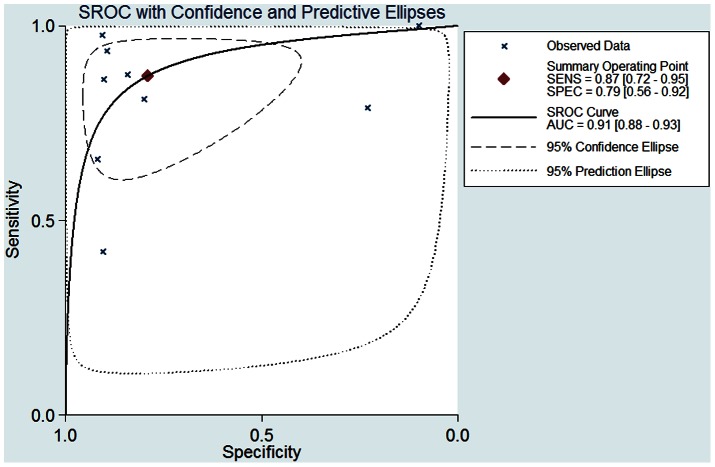
Summary receiver operating characteristic (SROC) curve for BALF sTREM-1 tests (9 studies). AUC = 0.91 (95% CI, 0.88–0.93), Q* = 0.83.

The SROC curve shows an overall summary of tests, which illustrates the relationship between SEN and SPE. As shown in Fig. 5, the area under the SROC curve was 0.91 (95% CI, 0.88–0.93) and the Q* was 0.83, indicating a high diagnostic accuracy.

**Figure 6:**
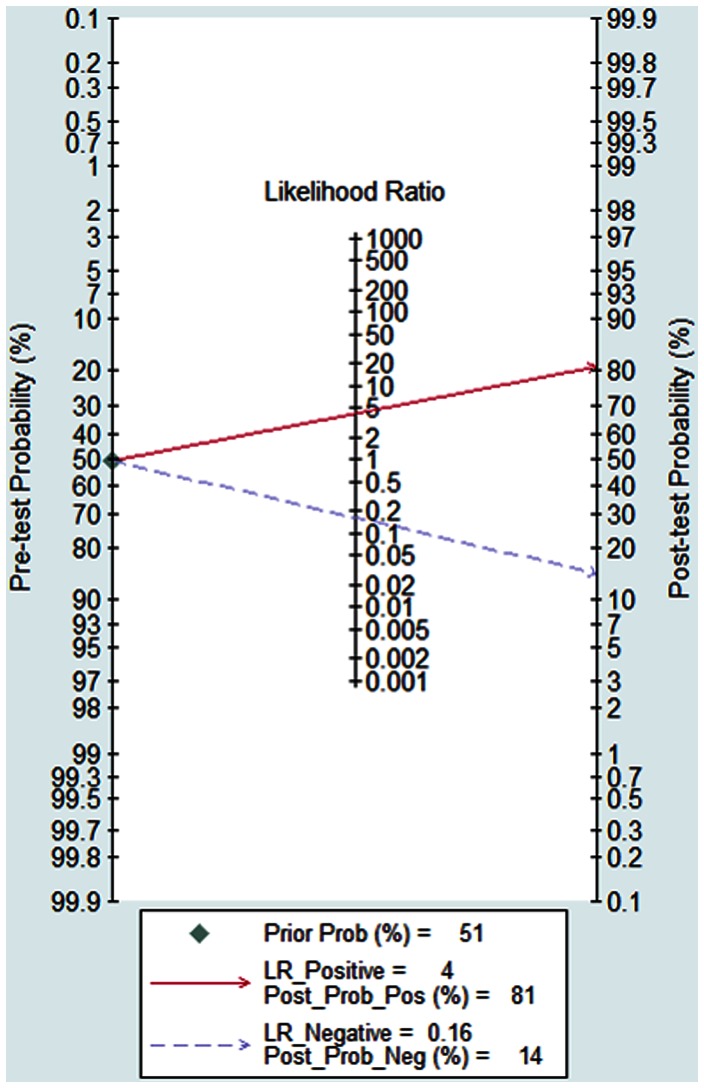
Fagan's nomogram for likelihood ratios and the probability for BALF sTREM-1 assays in the diagnosis of bacterial lung infections. The pre-test probability of disease was 51%.

Fig. 6 shows the Fagan's nomogram for likelihood ratios, and the results indicated that the BALF sTREM-1 for detection lung infections increased the post-probability to 81% when the results were positive and reduced the post-probability to 14% when the results were negative.

### 4. Heterogeneity assessment and meta-regression analysis

The I^2^ test for the pooled DOR indicated the I^2^ was 78%, and that was 84% and 90% for SEN and SPE, respectively, which showed substantial heterogeneity among studies.

**Figure 7:**
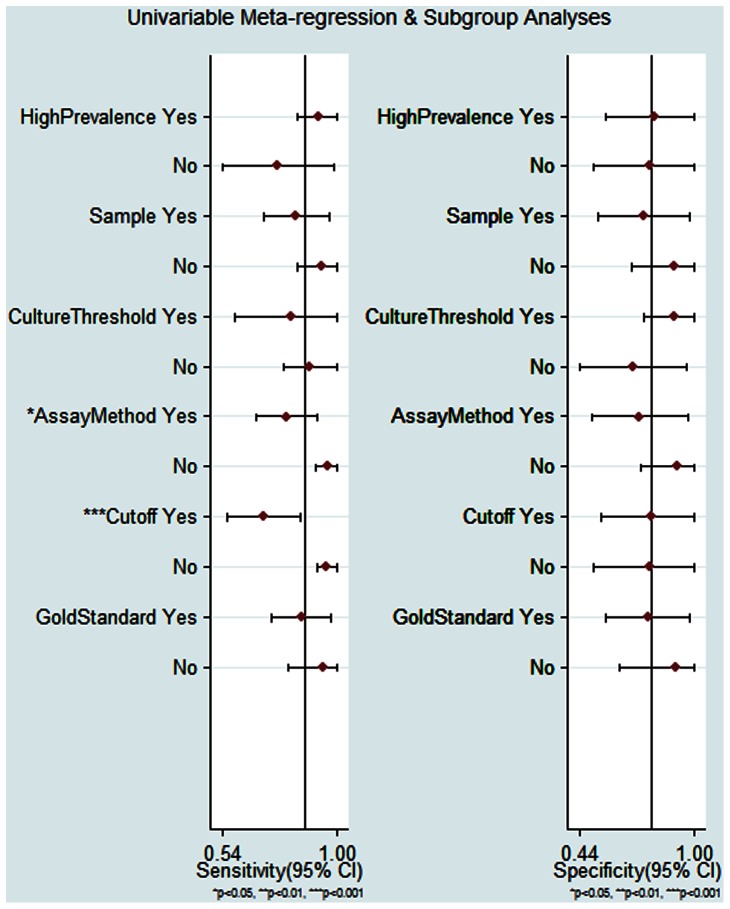
Forest plot of subgroup analysis for sensitivity and specificity. * *P*<0.05; ** *P*<0.01; *** *P*<0.001.

We chose to investigate whether the prevalence, sample, culture threshold, assay method, cutoff value and the gold standard were responsible for the heterogeneity. The meta-regression analysis showed that the assay method and cutoff value influenced the SEN, however, these covariates did not affect the SPE (Fig. 7).

### 5. Evaluation of publication bias

**Figure 8:**
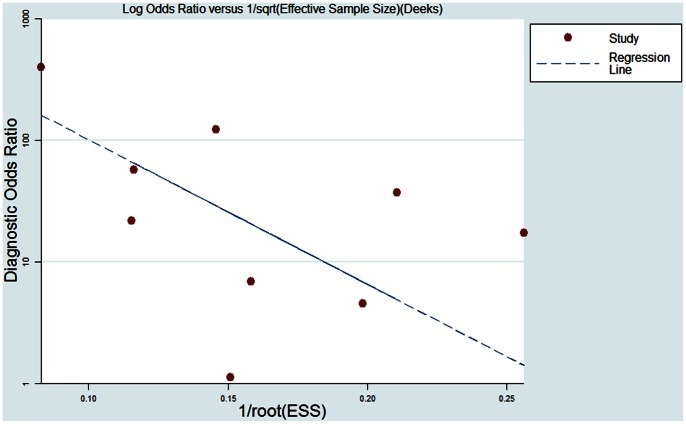
The Deek's funnel plot for the assessment of potential publication bias. There was no significant publication bias (*P* = 0.06).

The Deek's funnel plot asymmetry test showed there was no publication bias (*P* = 0.06) (Fig. 8) [14].

## Discussion

Lung infection is a common challenge for patients in the ICU. Earlier identification of lung infection and earlier treatment are very important for ICU patients. Although great advances have been made in the diagnosis and treatment of lung infection in critically ill patients, it is still necessary to find more accurate biomarker to diagnose lung infection in order to reduce the morbidity and mortality.

Triggering Receptors Expressed on Myeloid cells-1 (TREM-1) belongs to the immunoglobulin super family. TREM-1 expression is increased in the presence of extracellular bacteria, fungi or inflammatory mediators stimulation [27], [28]. In normal lung tissues, TREM-1 is selectively expressed on alveolar macrophages [27]. Soluble TREM-1 (sTREM-1) will be either secreted or shed under the condition of infection [29], and then sTREM-1 can be detected in BALF [26].

A previous study has evaluated the serum sTREM-1 in diagnosing bacterial infections in emergency department with a sensitivity of 82% and a specificity of 40% (with a cutoff value of 3.5 µg/L) [8]. It is obvious that the specificity in that study was very low. Ramirez et al. [9] recently reported that the BALF sTREM-1 seemed more accurate in diagnosing lung infections for ICU patients with a sensitivity of 81% and a specificity of 80% (with a cutoff value of 900 pg/ml). In this regard, we performed the meta-analysis to evaluate the accuracy of BALF sTREM-1 for diagnosing lung infections in ICU patients.

The present study revealed that the BALF sTREM-1 had a good diagnostic accuracy for bacterial lung infections in ICU patients. The area under the SROC curve was 0.91 and the Q* was 0.83. The pooled SEN of BALF sTREM-1 in diagnosing lung infections was 87% and the SPE was 79%. We also investigated LR and the results indicated that pooled PLR was 4.18 and the NLR was 0.16. With a pre-test probability of 51% the BALF sTREM-1 raised the post-test probability to 81% when the results were positive and reduced the post-probability to 14% when the results were negative, which showed the application of BALF sTREM-1 could be helpful in discriminating the bacterial lung infections. The DOR represents a single indicator of test accuracy [30]. The pooled DOR in the present meta-analysis was 25.60, which further confirmed BALF sTREM-1 was useful in discriminating the lung infections.

The I^2^ test for the pooled DOR, SEN and SPE showed there was marked heterogeneity between the studies. And then we undertook a meta-regression analysis to explore the possible reasons for the heterogeneity [31]. The subgroup analysis showed that assay method and cutoff value affected the diagnostic SEN of BALF sTREM-1 but had no effect on SPE. The pooled SEN of BALF sTREM-1 for the diagnosis of lung infection was higher in the studies using immunoblot technique assay method than that in the studies using ELISA. The immunoblot technique assay method was used in two studies by Gibot et al., although this method resulted a higher SEN, the test procedure seemed more complicated than ELISA, which might make it difficult to be applied widely. We also found that higher cutoff values led to lower SEN. We could not evaluate the optimized cutoff value of BALF sTREM-1 for diagnosing bacterial lung infection in ICU patients due to different cutoff values were used in the included studies. The subgroup analysis indicated the SEN and SPE of “clinical+radiological+microbiological” standard were comparable to that of “clinical+microbiological” standard, which implied that both standards were similar for the diagnosis of bacterial lung infection in ICU patients.

It should be noted that, there are some limitations in our meta-analysis. First, since the BALF quantitative culture was chosen as the diagnostic criteria, the use of antibiotics in some patients could probably influence the culture results, which would increase the number of false-negative patients. The SPE was 79% in this study, which indicated a 21% of false positive. As we know, the ICU patients could have different kinds of organs infections, central venous, urethral, or other catheters infections, which may influence the BALF sTREM-1 levels and lead to false positive. Nevertheless, most of the studies did not describe details about these factors. Second, most included studies did not describe the details of culture procedure, for example, whether anaerobic culture was performed. This would omit some infected patients. Third, different BALF samples and different cutoff values of BALF sTREM-1 were used in the included studies, which made it difficult to determine the optimized cutoff value. In order to further evaluate the diagnostic accuracy of BALF sTREM-1 for different types of bacterial lung infections in ICU patients, future studies which categorize the bacteria, describe the detailed culture method and procedure and use the same assay method for sTREM-1 (e.g., ELISA) are needed.
